# 3-(2-Hydroxy­ethyl)-2-(*p*-tolyl­amino)­quinazolin-4(3*H*)-one

**DOI:** 10.1107/S1600536808040440

**Published:** 2008-12-06

**Authors:** Gui-Fu Zhang, Zuan Ma, Xu-Hong Yang

**Affiliations:** a Department of Chemistry and Life Science, Xianning College, Xianning 4371000, Hubei, People’s Republic of China; b Department of Medicinal Chemistry, Yunyang Medical College, Shiyan 442000, Hubei, People’s Republic of China

## Abstract

In the title compound, C_17_H_17_N_3_O_2_, the quinazolinone ring system is essentially planar. The benzene ring is twisted with respect to it by a dihedral angle of 32.7 (5)°. The mol­ecular conformation is stabilized by an N—H⋯O hydrogen bond, and the crystal structure is stabilized by inter­molecular O—H⋯N inter­actions.

## Related literature

For the biological properties of quinazolinone derivatives, see: Pandeya *et al.* (1999 ▶); Shiba *et al.* (1997 ▶), Malamas & Millen (1991 ▶); Mannschreck *et al.* (1984 ▶); Kung *et al.* (1999 ▶); Bartroli *et al.* (1998 ▶); Palmer *et al.* (1997 ▶); Tsou *et al.* (2001 ▶); Matsuno *et al.* (2002 ▶). For the synthesis, see: Yang *et al.* (2008 ▶). For related structures, see: Hu *et al.* (2006 ▶); Qu *et al.* (2008 ▶); Zeng *et al.* (2008 ▶); Sun *et al.* (2008 ▶).
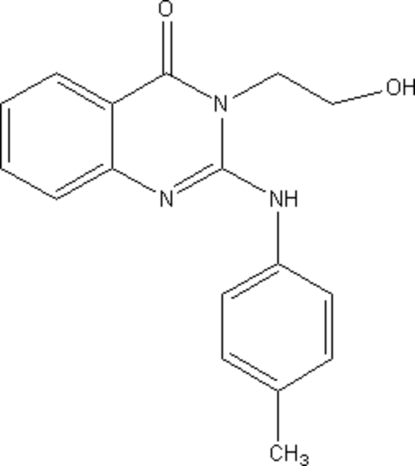


## Experimental

### 

#### Crystal data

C_17_H_17_N_3_O_2_
*M* *_r_* = 295.34
Monoclinic, 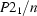

*a* = 7.8589 (2) Å
*b* = 19.1706 (5) Å
*c* = 10.6696 (3) Å
β = 111.082 (3)°
*V* = 1499.89 (8) Å^3^
*Z* = 4
Mo *K*α radiation
μ = 0.09 mm^−1^
*T* = 298 (2) K
0.10 × 0.10 × 0.08 mm

#### Data collection

Bruker SMART 4K CCD area-detector diffractometer
Absorption correction: multi-scan (*SADABS*; Sheldrick, 2001 ▶) *T*_min_ = 0.981, *T*_max_ = 0.993
15404 measured reflections
2938 independent reflections
2074 reflections with *I* > 2σ(*I*)
*R*_int_ = 0.037

#### Refinement

*R*[*F* ^2^ > 2σ(*F* ^2^)] = 0.046
*wR*(*F* ^2^) = 0.138
*S* = 1.07
2938 reflections
206 parameters
2 restraints
H atoms treated by a mixture of independent and constrained refinement
Δρ_max_ = 0.23 e Å^−3^
Δρ_min_ = −0.17 e Å^−3^

### 

Data collection: *SMART* (Bruker, 2000 ▶); cell refinement: *SAINT-Plus* (Bruker, 2000 ▶); data reduction: *SAINT-Plus*; program(s) used to solve structure: *SHELXS97* (Sheldrick, 2008 ▶); program(s) used to refine structure: *SHELXL97* (Sheldrick, 2008 ▶); molecular graphics: *PLATON* (Spek, 2003 ▶); software used to prepare material for publication: *SHELXTL* (Sheldrick, 2008 ▶).

## Supplementary Material

Crystal structure: contains datablocks global, I. DOI: 10.1107/S1600536808040440/bt2827sup1.cif

Structure factors: contains datablocks I. DOI: 10.1107/S1600536808040440/bt2827Isup2.hkl

Additional supplementary materials:  crystallographic information; 3D view; checkCIF report

## Figures and Tables

**Table 1:** Hydrogen-bond geometry (Å, °)

*D*—H⋯*A*	*D*—H	H⋯*A*	*D*⋯*A*	*D*—H⋯*A*
O1—H1*D*⋯N3^i^	0.88 (15)	2.09 (15)	2.882 (12)	149 (13)
N1—H1⋯O1	0.87 (7)	1.98 (8)	2.806 (12)	160 (12)

Symmetry code: (i) 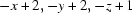.

**Fig. 1.**
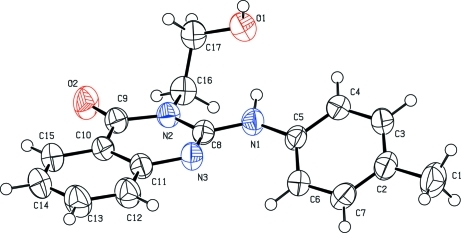
View of the molecular structure of the title compound, showing the atom labelling scheme. Displacement ellipsoids are drawn at the 50% probability level.

**Fig. 2.**
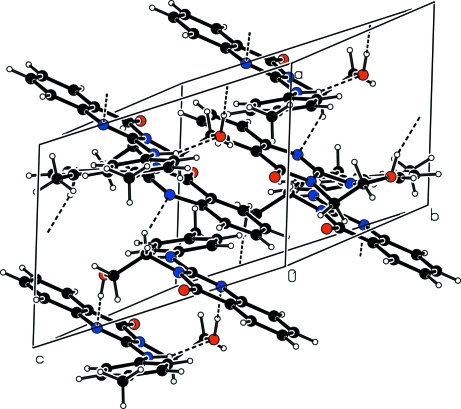
Crystal packing of the title compound, showing the hydrogen bonds as dashed lines.

## supplementary crystallographic information

### Comment

The synthesis of derivatives of quinazolinone has been the focus of great interest. This is due, in part, to the broad spectrum of biological properties of these compounds. Some of these activities include antimicrobial (Pandeya *et al.*, 1999; Shiba *et al.*, 1997), antidiabetic (Malamas & Millen, 1991), anticonvulsant (Mannschreck *et al.*, 1984), antibacterial (Kung *et al.*, 1999), antifungal (Bartroli *et al.*, 1998), protein tyrosine kinase inhibitors (Palmer *et al.*, 1997), EGFR inhibitors (Tsou *et al.*, 2001) and PDGFR phosphorylation inhibitors (Matsuno *et al.*, 2002). We have recently focused on the synthesis of heterocyclic compounds using an aza-Wittig reaction. The compound  (Fig. 1), may be used as a new precursor for obtaining bioactive molecules. The bond lengths and angles are unexceptional. The quinazolinone ring system is almost planar, with a maximum deviation of 0.037Å for N2; the   phenyl ring is twisted with respect to it, with a dihedral angle of 32.7 (5)°. Intramolecular N—H···O and intermolecular O—H···N   hydrogen bonds (Fig. 2 and Table 2) stabilize the molecular conformation and  the crystal structure.

### Experimental

To a solution of 1-(4-methyl-phenyl)- 3-(2-ethoxycarbonylphenyl) carbodiimide (3 mmol) in THF (15 ml) was added 2-aminoethanol (3 mmol). After the reaction mixture was allowed to stand for 1 h, the solvent was removed and anhydrous ethanol (10 ml) with several drops of EtONa in EtOH was added. The mixture was stirred for 4 h at room temperature. The solution was concentrated under reduced pressure and the residue was recrystallized from ethanol to give the title compound. The product was recrystallized from methanol-dichloromethane (1:1 *v*/*v*, 20 ml) at room temperature to give crystals suitable for X-ray diffraction.

### Refinement

All H atoms were located in difference maps. Those bonded to C were treated as riding atoms with C—H = 0.93 Å, *U*_iso_=1.2*U*_eq_ (C) for C*sp*^2^, C—H = 0.97 Å, *U*_iso_ = 1.2*U*_eq_ (C) for CH_2_. The coordinates of the H atoms bonded to N and O were refined with a distance restraint of  O—H = 0.88 (2)Å and *U*_iso_ = 1.2*U*_eq_ (O, N).

### Crystal data

**Table:**

C_17_H_17_N_3_O_2_	*F*(000) = 624
*M**_r_* = 295.34	*D*_x_ = 1.308 Mg m^−^^3^
Monoclinic, *P*2_1_/*n*	Mo *K*α radiation, λ = 0.71073 Å
Hall symbol: -P 2yn	Cell parameters from 2754 reflections
*a* = 7.8589 (2) Å	θ = 2.3–23.8°
*b* = 19.1706 (5) Å	µ = 0.09 mm^−^^1^
*c* = 10.6696 (3) Å	*T* = 298 K
β = 111.082 (3)°	Block, colorless
*V* = 1499.89 (8) Å^3^	0.10 × 0.10 × 0.08 mm
*Z* = 4	

### Data collection

**Table:**

Bruker SMART 4K CCD area-detector diffractometer	2938 independent reflections
Radiation source: fine-focus sealed tube	2074 reflections with *I* > 2σ(*I*)
graphite	*R*_int_ = 0.037
φ and ω scans	θ_max_ = 26.0°, θ_min_ = 2.1°
Absorption correction: multi-scan (*SADABS*; Sheldrick, 2001)	*h* = −9→9
*T*_min_ = 0.981, *T*_max_ = 0.993	*k* = −23→23
15404 measured reflections	*l* = −13→11

### Refinement

**Table:**

Refinement on *F*^2^	Primary atom site location: structure-invariant direct methods
Least-squares matrix: full	Secondary atom site location: difference Fourier map
*R*[*F*^2^ > 2σ(*F*^2^)] = 0.046	Hydrogen site location: inferred from neighbouring sites
*wR*(*F*^2^) = 0.138	H atoms treated by a mixture of independent and constrained refinement
*S* = 1.07	*w* = 1/[σ^2^(*F*_o_^2^) + (0.081*P*)^2^ + 0.012*P*] where *P* = (*F*_o_^2^ + 2*F*_c_^2^)/3
2938 reflections	(Δ/σ)_max_ < 0.001
206 parameters	Δρ_max_ = 0.23 e Å^−^^3^
2 restraints	Δρ_min_ = −0.17 e Å^−^^3^

### Special details

**Table:**

Geometry. All e.s.d.'s (except the e.s.d. in the dihedral angle between two l.s. planes) are estimated using the full covariance matrix. The cell e.s.d.'s are taken into account individually in the estimation of e.s.d.'s in distances, angles and torsion angles; correlations between e.s.d.'s in cell parameters are only used when they are defined by crystal symmetry. An approximate (isotropic) treatment of cell e.s.d.'s is used for estimating e.s.d.'s involving l.s. planes.
Refinement. Refinement of *F*^2^ against ALL reflections. The weighted *R*-factor *wR* and goodness of fit *S* are based on *F*^2^, conventional *R*-factors *R* are based on *F*, with *F* set to zero for negative *F*^2^. The threshold expression of *F*^2^ > σ(*F*^2^) is used only for calculating *R*-factors(gt) *etc*. and is not relevant to the choice of reflections for refinement. *R*-factors based on *F*^2^ are statistically about twice as large as those based on *F*, and *R*- factors based on ALL data will be even larger.

### Fractional atomic coordinates and isotropic or equivalent isotropic displacement parameters (Å^2^)

**Table:**

	*x*	*y*	*z*	*U*_iso_*/*U*_eq_	
C1	0.713 (2)	0.6964 (7)	0.4651 (16)	0.077 (5)	
H1A	0.5917	0.6810	0.4512	0.116*	
H1B	0.7975	0.6738	0.5432	0.116*	
H1C	0.7434	0.6846	0.3881	0.116*	
C2	0.7255 (16)	0.7741 (6)	0.4853 (13)	0.053 (3)	
C3	0.6939 (17)	0.8197 (6)	0.3785 (13)	0.056 (3)	
H3	0.6602	0.8019	0.2918	0.068*	
C4	0.7113 (16)	0.8908 (6)	0.3978 (11)	0.050 (3)	
H4	0.6892	0.9202	0.3243	0.060*	
C5	0.7611 (14)	0.9190 (5)	0.5253 (11)	0.042 (3)	
C6	0.7883 (15)	0.8745 (6)	0.6330 (12)	0.048 (3)	
H6	0.8185	0.8924	0.7193	0.058*	
C7	0.7703 (16)	0.8033 (6)	0.6116 (13)	0.053 (3)	
H7	0.7891	0.7740	0.6848	0.063*	
C8	0.8785 (14)	1.0302 (5)	0.6438 (11)	0.041 (3)	
C9	0.9600 (16)	1.1474 (6)	0.7315 (11)	0.047 (3)	
C10	1.0902 (15)	1.1136 (6)	0.8487 (11)	0.044 (3)	
C11	1.1021 (15)	1.0410 (6)	0.8529 (11)	0.044 (3)	
C12	1.2277 (17)	1.0094 (7)	0.9662 (12)	0.059 (3)	
H12	1.2365	0.9610	0.9711	0.070*	
C13	1.3379 (19)	1.0490 (8)	1.0699 (13)	0.066 (4)	
H13	1.4213	1.0272	1.1448	0.080*	
C14	1.3277 (19)	1.1213 (7)	1.0656 (12)	0.064 (4)	
H14	1.4040	1.1478	1.1366	0.077*	
C15	1.2045 (18)	1.1529 (7)	0.9560 (12)	0.057 (3)	
H15	1.1965	1.2013	0.9528	0.068*	
C16	0.7068 (16)	1.1345 (6)	0.5175 (11)	0.049 (3)	
H16A	0.6734	1.1787	0.5463	0.058*	
H16B	0.5999	1.1047	0.4912	0.058*	
C17	0.7589 (16)	1.1470 (6)	0.3967 (11)	0.051 (3)	
H17A	0.6786	1.1817	0.3390	0.061*	
H17B	0.8828	1.1645	0.4253	0.061*	
N1	0.7697 (13)	0.9925 (5)	0.5361 (9)	0.048 (3)	
H1	0.737 (16)	1.015 (6)	0.461 (9)	0.057*	
N2	0.8506 (12)	1.1022 (4)	0.6320 (9)	0.042 (2)	
N3	0.9966 (12)	0.9998 (5)	0.7464 (9)	0.046 (2)	
O1	0.7451 (12)	1.0832 (4)	0.3238 (8)	0.055 (2)	
H1D	0.84 (2)	1.074 (7)	0.306 (15)	0.083*	
O2	0.9394 (12)	1.2106 (4)	0.7163 (9)	0.064 (3)	

### Atomic displacement parameters (Å^2^)

**Table:**

	*U*^11^	*U*^22^	*U*^33^	*U*^12^	*U*^13^	*U*^23^
C1	0.090 (11)	0.048 (8)	0.099 (12)	−0.014 (7)	0.040 (10)	−0.019 (7)
C2	0.049 (7)	0.045 (7)	0.068 (8)	−0.010 (5)	0.025 (6)	−0.011 (6)
C3	0.064 (8)	0.056 (7)	0.054 (8)	−0.018 (6)	0.028 (7)	−0.018 (6)
C4	0.052 (7)	0.051 (7)	0.046 (7)	−0.011 (5)	0.018 (6)	−0.003 (5)
C5	0.037 (6)	0.040 (6)	0.046 (6)	−0.009 (4)	0.013 (5)	−0.003 (5)
C6	0.051 (7)	0.047 (7)	0.046 (6)	−0.010 (5)	0.016 (6)	−0.006 (5)
C7	0.052 (8)	0.045 (7)	0.059 (8)	−0.008 (5)	0.018 (6)	0.004 (5)
C8	0.041 (6)	0.037 (6)	0.044 (6)	−0.003 (5)	0.015 (5)	−0.004 (5)
C9	0.056 (7)	0.038 (6)	0.051 (7)	−0.001 (5)	0.025 (6)	−0.008 (5)
C10	0.049 (7)	0.041 (6)	0.043 (6)	−0.004 (5)	0.020 (5)	−0.007 (5)
C11	0.045 (7)	0.045 (6)	0.040 (6)	−0.003 (5)	0.013 (5)	−0.006 (5)
C12	0.066 (8)	0.052 (7)	0.047 (7)	0.001 (6)	0.007 (6)	0.000 (5)
C13	0.065 (9)	0.075 (9)	0.045 (7)	−0.002 (7)	0.002 (7)	−0.005 (6)
C14	0.070 (9)	0.070 (9)	0.044 (7)	−0.016 (7)	0.010 (7)	−0.015 (6)
C15	0.069 (9)	0.049 (7)	0.052 (7)	−0.011 (6)	0.022 (7)	−0.014 (6)
C16	0.043 (7)	0.044 (6)	0.055 (7)	0.009 (5)	0.013 (6)	0.001 (5)
C17	0.052 (7)	0.043 (6)	0.047 (7)	0.005 (5)	0.007 (6)	0.003 (5)
N1	0.052 (6)	0.040 (5)	0.041 (5)	−0.005 (4)	0.006 (5)	0.000 (4)
N2	0.043 (5)	0.037 (5)	0.044 (5)	0.003 (4)	0.014 (4)	−0.001 (4)
N3	0.050 (6)	0.037 (5)	0.043 (5)	−0.001 (4)	0.007 (5)	−0.003 (4)
O1	0.060 (6)	0.053 (5)	0.049 (5)	0.005 (4)	0.015 (4)	−0.003 (4)
O2	0.083 (7)	0.035 (5)	0.069 (6)	0.005 (4)	0.020 (5)	−0.006 (4)

### Geometric parameters (Å, °)

**Table:**

C1—C2	1.505 (17)	C10—C11	1.394 (15)
C1—H1A	0.9600	C10—C15	1.394 (15)
C1—H1B	0.9600	C11—N3	1.388 (14)
C1—H1C	0.9600	C11—C12	1.395 (16)
C2—C7	1.382 (17)	C12—C13	1.365 (17)
C2—C3	1.385 (18)	C12—H12	0.9300
C3—C4	1.380 (16)	C13—C14	1.387 (19)
C3—H3	0.9300	C13—H13	0.9300
C4—C5	1.383 (15)	C14—C15	1.363 (18)
C4—H4	0.9300	C14—H14	0.9300
C5—C6	1.384 (15)	C15—H15	0.9300
C5—N1	1.414 (13)	C16—N2	1.470 (14)
C6—C7	1.383 (15)	C16—C17	1.506 (16)
C6—H6	0.9300	C16—H16A	0.9700
C7—H7	0.9300	C16—H16B	0.9700
C8—N3	1.292 (14)	C17—O1	1.432 (13)
C8—N1	1.366 (14)	C17—H17A	0.9700
C8—N2	1.395 (13)	C17—H17B	0.9700
C9—O2	1.225 (13)	N1—H1	0.87 (7)
C9—N2	1.401 (14)	O1—H1D	0.88 (15)
C9—C10	1.452 (16)		
			
C2—C1—H1A	109.5	N3—C11—C12	119.3 (10)
C2—C1—H1B	109.5	C10—C11—C12	118.7 (10)
H1A—C1—H1B	109.5	C13—C12—C11	120.4 (12)
C2—C1—H1C	109.5	C13—C12—H12	119.8
H1A—C1—H1C	109.5	C11—C12—H12	119.8
H1B—C1—H1C	109.5	C12—C13—C14	121.1 (12)
C7—C2—C3	117.0 (11)	C12—C13—H13	119.5
C7—C2—C1	121.4 (12)	C14—C13—H13	119.5
C3—C2—C1	121.5 (12)	C15—C14—C13	119.2 (11)
C4—C3—C2	121.4 (11)	C15—C14—H14	120.4
C4—C3—H3	119.3	C13—C14—H14	120.4
C2—C3—H3	119.3	C14—C15—C10	120.8 (12)
C3—C4—C5	120.7 (11)	C14—C15—H15	119.6
C3—C4—H4	119.6	C10—C15—H15	119.6
C5—C4—H4	119.6	N2—C16—C17	114.5 (9)
C4—C5—C6	118.7 (10)	N2—C16—H16A	108.6
C4—C5—N1	117.2 (10)	C17—C16—H16A	108.6
C6—C5—N1	123.9 (10)	N2—C16—H16B	108.6
C7—C6—C5	119.6 (11)	C17—C16—H16B	108.6
C7—C6—H6	120.2	H16A—C16—H16B	107.6
C5—C6—H6	120.2	O1—C17—C16	109.8 (9)
C2—C7—C6	122.4 (11)	O1—C17—H17A	109.7
C2—C7—H7	118.8	C16—C17—H17A	109.7
C6—C7—H7	118.8	O1—C17—H17B	109.7
N3—C8—N1	121.1 (10)	C16—C17—H17B	109.7
N3—C8—N2	124.4 (9)	H17A—C17—H17B	108.2
N1—C8—N2	114.6 (9)	C8—N1—C5	126.3 (9)
O2—C9—N2	119.7 (11)	C8—N1—H1	113 (9)
O2—C9—C10	125.0 (10)	C5—N1—H1	116 (8)
N2—C9—C10	115.3 (10)	C8—N2—C9	120.6 (9)
C11—C10—C15	119.8 (11)	C8—N2—C16	122.7 (9)
C11—C10—C9	119.4 (9)	C9—N2—C16	116.6 (9)
C15—C10—C9	120.8 (11)	C8—N3—C11	118.2 (9)
N3—C11—C10	121.9 (10)	C17—O1—H1D	113 (10)
			
C7—C2—C3—C4	1.7 (18)	C13—C14—C15—C10	0(2)
C1—C2—C3—C4	−177.7 (11)	C11—C10—C15—C14	−0.2 (18)
C2—C3—C4—C5	0.0 (18)	C9—C10—C15—C14	−179.5 (11)
C3—C4—C5—C6	−1.8 (17)	N2—C16—C17—O1	79.0 (12)
C3—C4—C5—N1	−177.9 (10)	N3—C8—N1—C5	4.7 (17)
C4—C5—C6—C7	1.8 (16)	N2—C8—N1—C5	−175.6 (9)
N1—C5—C6—C7	177.6 (10)	C4—C5—N1—C8	−152.1 (11)
C3—C2—C7—C6	−1.7 (18)	C6—C5—N1—C8	32.0 (17)
C1—C2—C7—C6	177.7 (11)	N3—C8—N2—C9	3.5 (15)
C5—C6—C7—C2	−0.1 (17)	N1—C8—N2—C9	−176.3 (9)
O2—C9—C10—C11	−179.1 (10)	N3—C8—N2—C16	−175.9 (10)
N2—C9—C10—C11	2.0 (14)	N1—C8—N2—C16	4.3 (14)
O2—C9—C10—C15	0.2 (17)	O2—C9—N2—C8	176.4 (10)
N2—C9—C10—C15	−178.7 (10)	C10—C9—N2—C8	−4.6 (14)
C15—C10—C11—N3	−177.3 (10)	O2—C9—N2—C16	−4.1 (15)
C9—C10—C11—N3	2.0 (15)	C10—C9—N2—C16	174.9 (9)
C15—C10—C11—C12	0.8 (16)	C17—C16—N2—C8	−84.7 (12)
C9—C10—C11—C12	−179.9 (10)	C17—C16—N2—C9	95.9 (11)
N3—C11—C12—C13	177.4 (12)	N1—C8—N3—C11	−179.6 (9)
C10—C11—C12—C13	−0.7 (18)	N2—C8—N3—C11	0.7 (16)
C11—C12—C13—C14	0(2)	C10—C11—N3—C8	−3.4 (16)
C12—C13—C14—C15	0(2)	C12—C11—N3—C8	178.5 (10)

### Hydrogen-bond geometry (Å, °)

**Table:**

*D*—H···*A*	*D*—H	H···*A*	*D*···*A*	*D*—H···*A*
O1—H1D···N3^i^	0.88 (15)	2.09 (15)	2.882 (12)	149 (13)
N1—H1···O1	0.87 (7)	1.98 (8)	2.806 (12)	160 (12)

Symmetry codes: (i) −*x*+2, −*y*+2, −*z*+1.
